# Supplementary material for: Plk4 Is a Novel Substrate of Protein Phosphatase 5
Source: Int J Mol Sci. 2023 Jan 19;24(3):2033. doi: 10.3390/ijms24032033 (PMC9917060; doi:10.3390/ijms24032033)
Supplement: Supplementary file 1 [file ijms-24-02033-s001.zip › Supplementary material_proof.pdf]

SUPPLEMENTARY MATERIAL

Plk4 Is a Novel Substrate of Protein Phosphatase 5

Edit Ábrahám [1], Zsuzsánna Réthi-Nagy [1,2], Péter Vilmos [3], Rita Sinka [4] and Zoltán Lipinszki [1,*]

[1] Biological Research Centre, Institute of Biochemistry, MTA SZBK Lendület Laboratory of Cell Cycle Regulation, ELKH, H-6726 Szeged, Hungary; abraham.edit@brc.hu (E.Á.); nagy.zsuzsanna@brc.hu (Z.R.-N.)
[2] Doctoral School of Biology, Faculty of Science and Informatics, University of Szeged, H-6726 Szeged, Hungary
[3] Biological Research Centre, Institute of Genetics, ELKH, H-6726 Szeged, Hungary; vilmos.peter@brc.hu (P.V.)
[4] Department of Genetics, University of Szeged, H-6726 Szeged, Hungary; rsinka@bio.u-szeged.hu (R.S.)
* Correspondence: lipinszki.zoltan@brc.hu (Z.L.)

Plk4 is a novel substrate of Protein Phosphatase 5

Edit Ábrahám[1], Zsuzsánna Réthi-Nagy[1,2], Péter Vilmos[3], Rita Sinka[4] and Zoltán Lipinszki[1,*]

[1] Biological Research Centre, Institute of Biochemistry, MTA SZBK Lendület Laboratory of Cell Cycle Regulation, ELKH, H-6726 Szeged, Hungary; abraham.edit@brc.hu (E.A.)
[2] Doctoral School of Biology, Faculty of Science and Informatics, University of Szeged, H-6726 Szeged, Hungary; nagy.zsuzsanna@brc.hu (Z.R.-N.)
[3] Biological Research Centre, Institute of Genetics, ELKH, H-6726 Szeged, Hungary; vilmos.peter@brc.hu (P.V.)
[4] Department of Genetics, University of Szeged, H-6726 Szeged, Hungary; rsinka@bio.u-szeged.hu (R.S.)
* Correspondence: lipinszki.zoltan@brc.hu (Z.L.)

Figure S1. Preparation and activity testing of wild type and mutant forms of PP5.

(**a**) Multiple sequence alignment of the *Drosophila* (Uniprot: Q9VH81) and human (Uniprot: P53041) Protein phosphatase 5 orthologues was performed by Clustal Omega (https://www.ebi.ac.uk/Tools/msa/clustalo/). The activator mutations (E97Q in DmPP5, E76Q in HsPP5) are highlighted in yellow, while the inactivator mutations (H326N in DmPP5, H304Q in HsPP5) are highlighted in green. (**b**) Schematic representation of the *Drosophila* (DmPP5) and human (HsPP5) PP5 proteins. Numbers show the amino acid endpoints of the indicated motifs and domains. Arrowheads indicate the introduced mutations. (**c**) Purified GST (negative control), and *Drosophila* GST-PP5, GST-PP5^{E97Q} and GST-PP5^{H326N} were subjected to pNPP dephosphorylation assay. The relative phosphatase activity of the proteins was followed by absorbance measurements at 405 nm.

```
DmPP5      MSSSELEVQKAADCQQEAKVPASVEITGSKQPEEDTNARTKAELDFAAAEQYKNQGNEML     60
HsPP5      MA---MAEGERTECA---------------EPP---RDEPPADGALKRAEELKTQANDYF     39
           *:   :   : ::*                :*    . .  *:  :  **: *.*.*: :

                                               97
DmPP5      KTKEFSKAIDMYTKAIELHPNSAIYYANRSLAHLRQESFGFALQDGVSAVKADPAYLKGY     120
HsPP5      KAKDYENAIKFYSQAIELNPSNAIYYGNRSLAYLRTECYGYALGDATRAIELDKKYIKGY     99
           *:*::.:**.:*::****:*..****.*****:** *.:*:** *.. *:: *  *:***
                                               76
DmPP5      YRRAAAHMSLGKFKQALCDFEFVAKCRPNDKDAKLKFTECNKIVKMRAFERAIAVDKPEK     180
HsPP5      YRRAASNMALGKFRAALRDYETVVKVKPHDKDAKMKYQECNKIVKQKAFERAIAGDEHKR     159
           *****::*:****: ** *:* *.* :*:*****:*: ******* :******* *: ::

DmPP5      TLSEMYSDMENITIEDDYKGPQLEDGKVTLKFMKELMEHYKAQKRLHRKFAYKILCEIDT     240
HsPP5      SVVD-SLDIESMTIEDEYSGPKLEDGKVTISFMKELMQWYKDQKKLHRKCAYQILVQVKE     218
           :: :   *:*.:****:*.**:*******:.******: ** **:**** **:** ::.

DmPP5      YMRAQPSLVDITVPDEEKFTICGDIHGQFYDLMNIFEINGLPSEKNPYLFNGDFVDRGSF     300
HsPP5      VLSKLSTLVETTLKETEKITVCGDTHGQFYDLLNIFELNGLPSETNPYIFNGDFVDRGSF     278
            :    :**: *: : **:*:*** *******:****:******.***:***********

                                   326
DmPP5      SVECIFTLFGFKLLYPNHFFLARGNHESINMNQMYGFTGEVTAKYTSAMADIFTQVFNWL     360
HsPP5      SVEVILTLFGFKLLYPDHFHLLRGNHETDNMNQIYGFEGEVKAKYTAQMYELFSEVFEWL     338
           *** *:**********:**.* *****: ****:*** ***.****: * ::*::**:**
                                   304
DmPP5      PLCHCINQKILVMHGGLFSTEDVTLDHIRRIERNCQPPEEGLMCELLWSDPQQWMGLGQS     420
HsPP5      PLAQCINGKVLIMHGGLFSEDGVTLDDIRKIERNRQPPDSGPMCDLLWSDPQPQNGRSIS     398
           **.:*** *:*:******* :.****.**:**** ***:.* **:*******   * . *

DmPP5      KRGVGIQFGPDVTEKFCKDNNLDYIIRSHEVKDMGYEVAHNGKCITVFSAPNYCDTMGNM     480
HsPP5      KRGVSCQFGPDVTKAFLEENNLDYIIRSHEVKAEGYEVAHGGRCVTVFSAPNYCDQMGNK     458
           ****. *******: * ::*************  ******.*:*:********** ***

DmPP5      GAFITITGNNLKPNYKSFEAVPHPDVKPMAYANSLMNWLA-       520
HsPP5      ASYIHLQGSDLRPQFHQFTAVPHPNVKPMAYANTLLQLGMM       499
           .::* : *.:*:*:::.* *****:********:*::
```

(a)

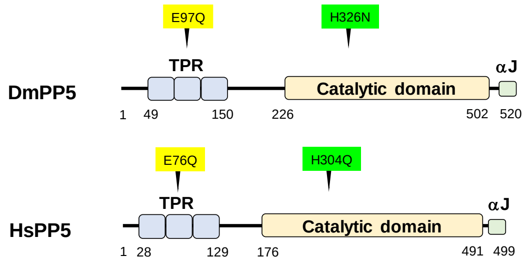

(b)

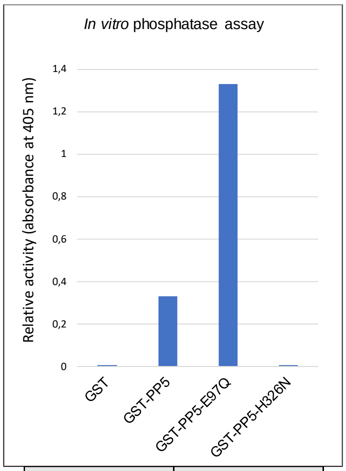

Proteins	Activity
GST	inactive
GST-PP5	active wild type
GST-PP5^{E97Q}	hyperactive
GST-PP5^{H326N}	inactive

(c)

Figure S2. Amounts of bait proteins used in the GST-IVTT binding assay.
Coomassie Brilliant Blue-stained SDS-PAGE gels corresponding to autoradiographs shown in Figure 2b were scanned before drying and autoradiography. Gels scans show the amount of bait (GST and GST-PP5) proteins used in *in vitro* binding assay. Asterisks indicate the abundant globin protein present in the IVTT inputs of the reticulocyte lysate.

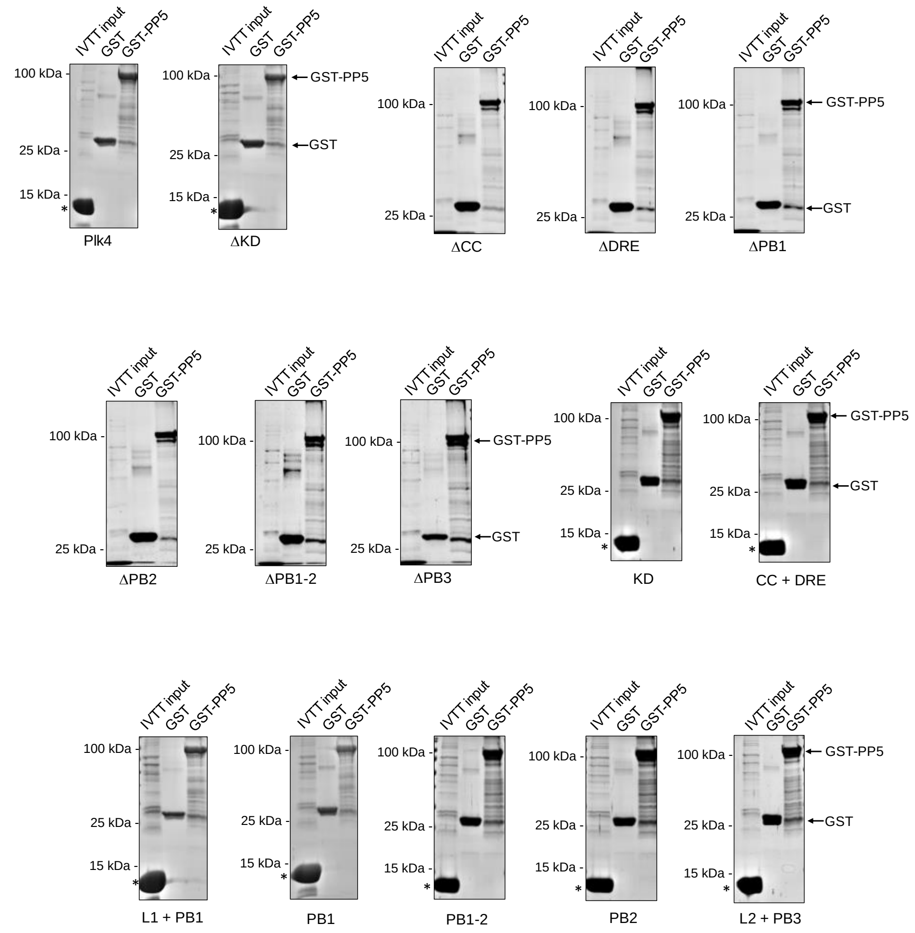

Figure S3. *In vivo* dephosphorylation of Plk4 in *Drosophila* cultured cells was tested by gel-shift assay.

GFP-Plk4-ND ("ND" refers to the non-degradable form of Plk4) or its kinase dead (NDKD) version were co-expressed with Myc-PP5^{E97Q} (hyperactive) or Myc-PP5^{H326N} (inactive), respectively, in D.Mel-2 cultured cells. Crude cell lysates were analysed by western blotting. In the presence of the active Myc-PP5^{E97Q}, GFP-Plk4-ND appeared as a discrete sharp band, similarly to the NDKD version, suggesting that it is dephosphorylated. The fuzzy and smeary band of GFP-Plk4-ND (indicated by arrowhead), however, indicates that it remained phosphorylated in the presence of the inactive Myc-PP5^{H326N}. Immunoblot (lower panel) with anti-PP5 shows equal amounts of the Myc-tagged PP5 derivatives (marked by asterisk) and endogenous PP5 as the loading control.

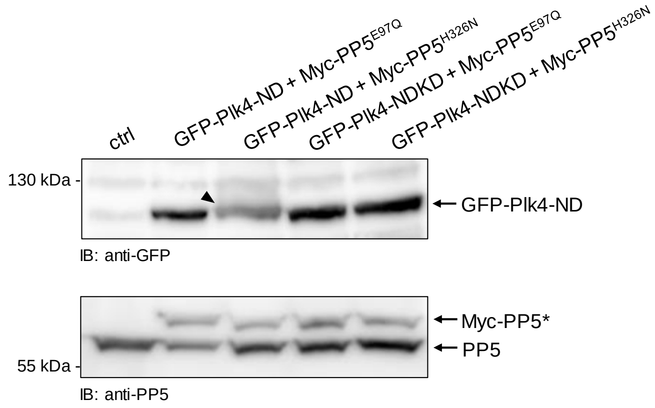

Figure S4. The phospho-status of Plk4 affects its binding to centrosomal proteins, *in vitro*. MBP-Plk4-TE co-expressed either with His$_6$-PP5 (active) or His$_6$-PP5^{H326N} (inactive) in bacteria were immobilized to amylose resin and incubated with ^{35}S-labelled Cep135, CP110, Sas4, Polo, Slimb, Ana1, Ana2, Sas6 and Spd2, respectively. The *in vitro* binding assay shows that in some cases the PP5-dephosphorylated MBP-Plk4-TE binds stronger to its partners (Cep135, CP110 and Sas4), while in other cases the dephosphorylation weakens/inhibits the binding or the interaction requires self-phosphorylated Plk4 (Polo, Slimb and Ana1). Ana2, Sas6 and Spd2 do not bind to either form of Plk4. MBP was used as negative control. "p" refers to phosphorylated protein.

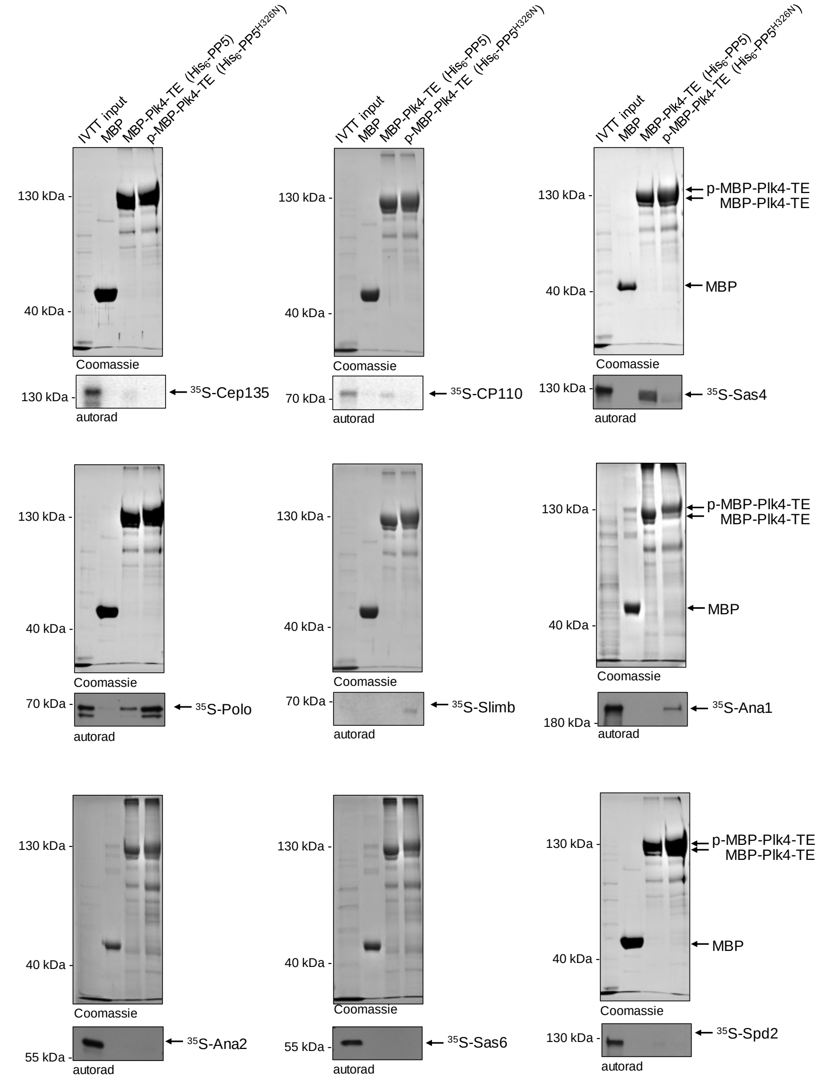

Figure S5. Validation of the anti-PP5 antibody and the efficiency of the RNAi.
(**a**) Whole body lysates of wild type (*w1118*), $pp5^{\Delta}$/+ heterozygote and two null mutant ($pp5^{\Delta}$/*Df(3R)5454 and* $pp5^{\Delta}$/*Df(3R)5428)* hemizygotic adult flies were analysed by western blotting using anti-PP5 polyclonal antibody. This reveals that the newly generated antibody recognizes endogenous PP5 around 60 kDa (as expected) and that PP5 disappears in the null mutants. Asterisk indicates a non-specific band that serves as the loading control. (**b**) PP5 was depleted from D.Mel-2 cultured cells by RNAi using dsRNA targeting the coding sequence (CDS) or the 3′ untranslated region (3′ UTR) of the endogenous *pp5* transcript. The immunoblot shows efficient knock-down of *pp5* (the level of the PP5 protein is decreased in both cases). Alpha tubulin (αTub) serves as the loading control (bottom panel). "ctrl" refers to control dsRNA treatment.

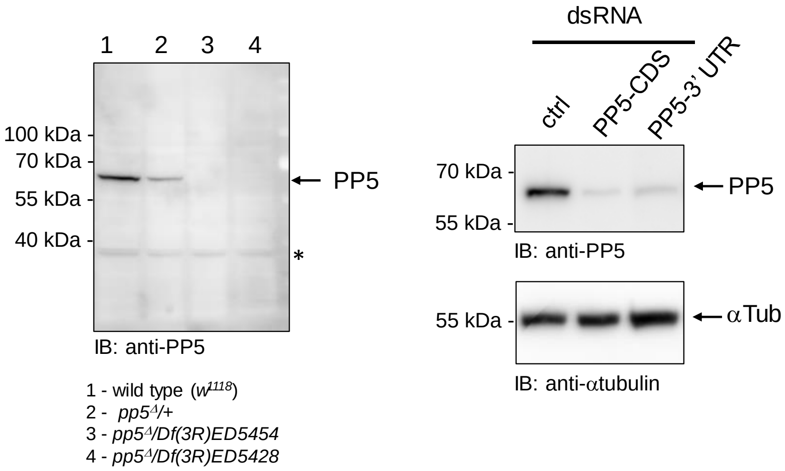

(a) (b)

Figure S6. Protein levels of PP5 and Plk4 in *Drosophila* ontogenetic stages and dissected tissues. (**a**) Protein levels of the endogenous PP5 and Plk4 were analysed by immunoblotting in samples collected from representative developmental stages of wild type fruit flies: 0-24 h: embryonal stages; L1-L3: larval stages; w.p.: white (early) pupa stage; b.p.: brown pupa stage; ph.a.: pharate adult (late pupa) stage; male and female adults. The image shows that PP5 is present throughout the ontogenesis, however, it is predominant during the embryogenesis. Plk4 can be observed mainly in early embryonic stages (i.e. in 0-2 h-old syncytial embryos). Actin5c immunoblot and the PonceauS-stained membrane of the anti-Plk4 blot serve as the loading control. (**b**) Protein levels of the endogenous PP5 and Plk4 were analysed by immunoblotting in syncytial embryos (0-2 h-old) and dissected ovaries or testes of wild type fruit flies. While PP5 is present in all tissues, Plk4 is exclusive to the ovaries (it appears in the embryos, too, which is due to maternal effect). Alpha tubulin (αTub) and the PonceauS-stained membrane of the anti-Plk4 blot serve as the loading control.

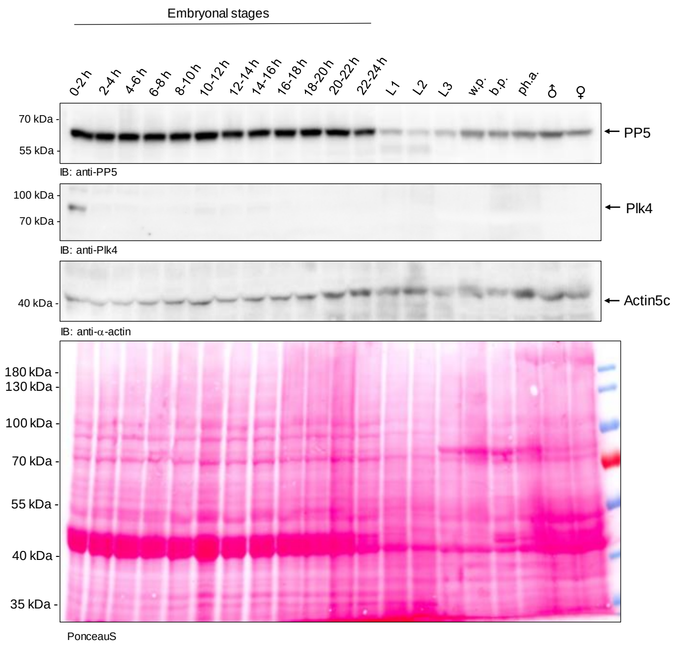

(a)

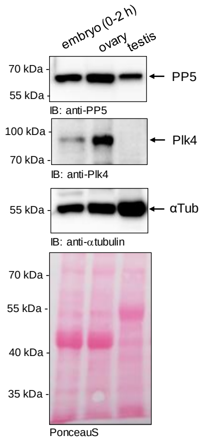

(b)

Figure S7. Negative controls corresponding to microscopic images shown in Figure 7.

(**a**) Representative stills of live cell imaging of D.Mel-2 cells expressing mCherry-Sas6 show that the co-expressed NYFP-Plk4 and CYFP (negative control) or NYFP and CYFP-PP5 (negative control), respectively, do not generate green signals co-localizing with centrosomes. Hoechst 33342 was used to visualize DNA. Scale bar: 10 µm. (**b**) Representative microscopic images of fixed D.Mel-2 cells show that the co-expressed NYFP-Plk4 and CYFP (negative control) or NYFP and CYFP-PP5 (negative control), respectively, do not generate specific green signals (dots). We counted $n = 100$ transfected cells and found that 0% of Asl dots show co-localisation with green (non-specific) signals. Anti-Asl in far red was used to visualize centrosomes. DAPI was used to visualize DNA. Scale bar: 10 µm.

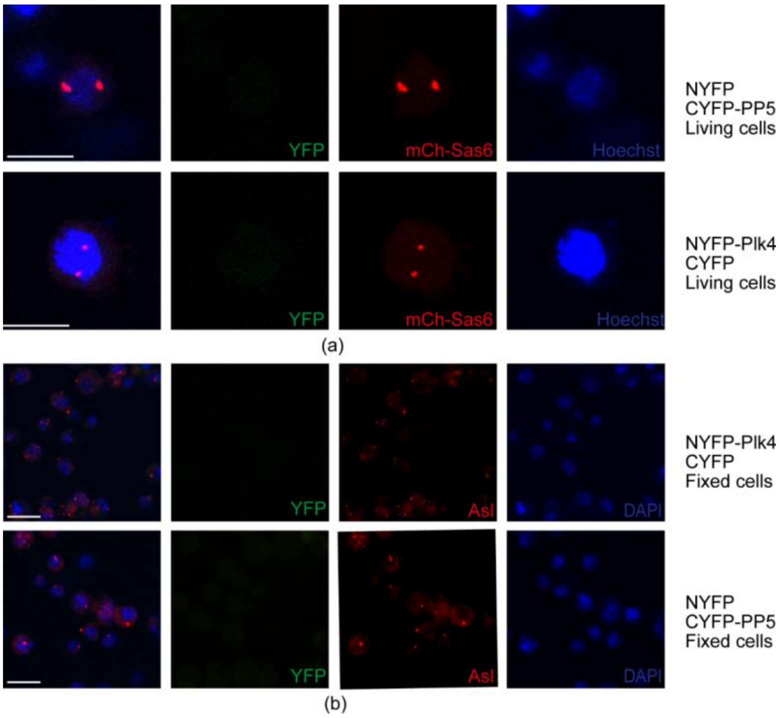

Figure S8. Basal body formation and testis morphology are normal in *pp5*$^{\Delta}$ mutant flies.

The GFP-tagged PACT domain (GFP-PACT) of the *Drosophila* pericentrin-like protein (d-Plp) was used to investigate the role of PP5 in spermatogenesis and basal body formation. The images show that GFP-PACT localizes normally to the basal body (indicated by arrowheads) both in wild type (GFP-PACT, first and second rows) and *pp5*$^{\Delta}$*/Df(3R)5454* (*pp5*$^{\Delta}$*/Df*, third and fourth rows) mutant testes. Normal nuclear (star) and cyst elongation, and also individualization complex (indicated by arrows) formation are observable in *pp5*$^{\Delta}$*/Df(3R)5454* males, similar to the wild type. Scale bar: 100 µm (10x objective, first and third rows) or 20 µm (60x objective, second and fourth rows). DAPI stains DNA and Phalloidin stains actin filaments.

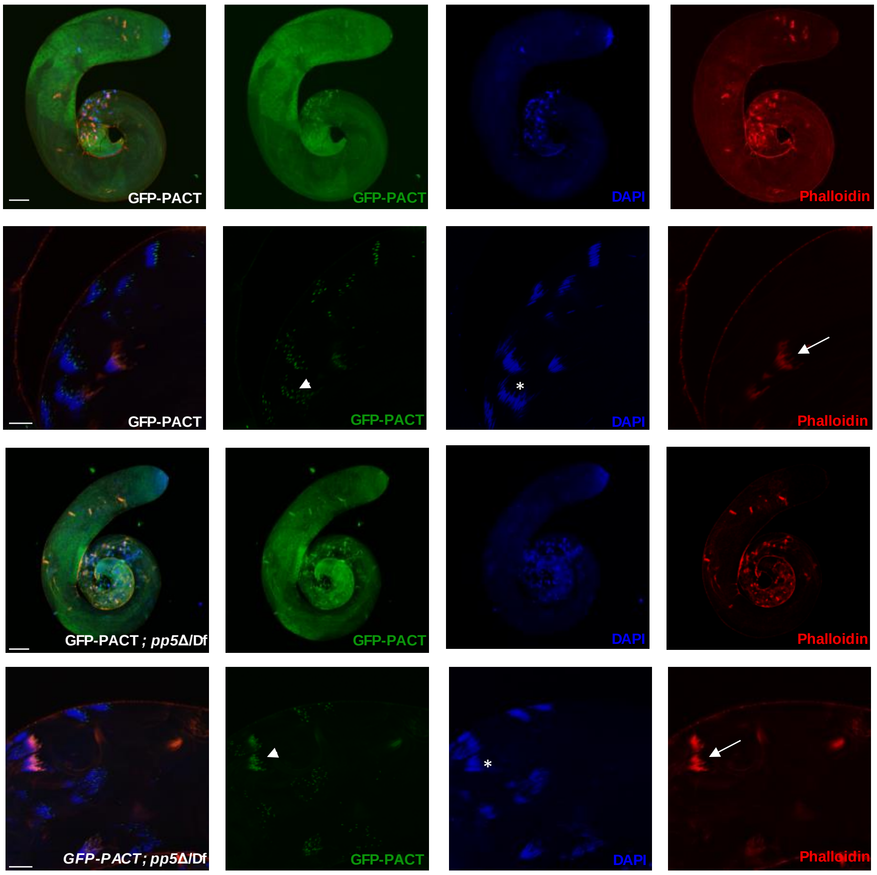

Figure S9. Sequences and maps of pMT(Hygro)-NYFP-Myc-GW, pMT(Hygro)-CYFP-HA-GW and pMT(Hygro)-mCherry-GW plasmids.

> pMT(Hygro)-NYFP-Myc-GW

TCGCGCGTTTCGGTGATGACGGTGAAAACCTCTGACACATGCAGCTCCCGGAGACGGTCACAGCTTGTCTGTAAGCGGATGCCGGGAGCAGACA
AGCCCGTCAGGGCGCGTCAGCGGGTGTTGGCGGGTGTCGGGGCTGGCTTAACTATGCGGCATCAGAGCAGATTGTACTGAGAGTGCACCATATG
CGGTGTGAAATACCGCACAGATGCGTAAGGAGAAAATACCGCATCAGGCGCCATTCGCCATTCAGGCTGCGCAACTGTTGGGAAGGGCGATCGG
TGCGGGCCTCTTCGCTATTACGCCAGCTGGCGAAAGGGGGATGTGCTGCAAGGCGATTAAGTTGGGTAACGCCAGGGTTTTCCCAGTCACGACG
TTGTAAAACGACGGCCAGTGCCAGTGAATTAATTCGTTGCAGGACAGGATGTGGTGCCCGATGTGACTAGCTCTTTGCTGCAGGCCGTCCTATC
CTCTGGTTCCGATAAGAGACCCAGAACTCCGGCCCCCCACCGCCCACCGCCACCCCCATACATATGTGGTACGCAAGTAAGAGTGCCTGCGCAT
GCCCCATGTGCCCCACCAAGAGTTTTGCATCCCATACAAGTCCCCAAAGTGGAGAACCGAACCAATTCTTCGCGGGCAGAACAAAAGCTTCTGC
ACACGTCTCCACTCGAATTTGGAGCCGGCCGGCGTGTGCAAAAGAGGTGAATCGAACGAAAGACCCGTGTGTAAAGCCGCGTTTCCAAAATGTA
TAAAACCGAGAGCATCTGGCCAATGTGCATCAGTTGTGGTCAGCAGCAAAATCAAGTGAATCATCTCAGTGCAACTAAAGGGGGGATCTAGATC
GGGGTACCACTGCAGTGAATTCGGAGaattcATGGTGAGCAAGGGCGAGGAGCTGTTCACCGGGGTGGTGCCCATCCTGGTCGAGCTGGACGGC
GACGTAAACGGCCACAAGTTCAGCGTGTCCGGCGAGGGCGAGGGCGATGCCACCTACGGCAAGCTGACCCTGAAGTTCATCTGCACCACCGGCA
AGCTGCCCGTGCCCTGGCCCACCCTCGTGACCACCTTCGGCTACGGCCTGCAGTGCTTCGCCCGCTACCCCGACCACATGAAGCAGCACGACTT
CTTCAAGTCCGCCATGCCCGAAGGCTACGTCCAGGAGCGCACCATCTTCTTCAAGGACGACGGCAACTACAAGACCCGCGCCGAGGTGAAGTTC
GAGGGCGACACCCTGGTGAACCGCATCGAGCTGAAGGGCATCGACTTCAAGGAGGACGGCAACATCCTGGGGCACAAGCTGGAGTACAACTACA
ACAGCCACAACGTCTATATCATGGCCGACAAGCAGAAGAACGGCATCAAGGTGAACTTCAAGATCCGCCACAACATCGAGGAGCAAAAGTTGAT
TTCTGAGGAGGATCTTtctagATCAACAAGTTTGTACAAAAAAGCTGAACGAGAAACGTAAAATGATATAAATATCAATATATTAAATTAGATT
TTGCATAAAAAACAGACTACATAATACTGTAAAACACAACATATCCAGTCACTATGGCGGCCGCATTAGGCACCCCAGGCTTTACACTTTATGC
TTCCGGCTCGTATAATGTGTGGATTTTGAGTTAGGATCCGTCGAGATTTTCAGGAGCTAAGGAAGCTAAAATGGAGAAAAAAATCACTGGATAT
ACCACCGTTGATATATCCCAATGGCATCGTAAAGAACATTTTGAGGCATTTCAGTCAGTTGCTCAATGTACCTATAACCAGACCGTTCAGCTGG
ATATTACGGCCTTTTTAAAGACCGTAAAGAAAAATAAGCACAAGTTTTATCCGGCCTTTATTCACATTCTTGCCCGCCTGATGAATGCTCATCC
GGAATTCCGTATGGCAATGAAAGACGGTGAGCTGGTGATATGGGATAGTGTTCACCCTTGTTACACCGTTTTCCATGAGCAAACTGAAACGTTT
TCATCGCTCTGGAGTGAATACCACGACGATTTCCGGCAGTTTCTACACATATATTCGCAAGATGTGGCGTGTTACGGTGAAAACCTGGCCTATT
TCCCTAAAGGGTTTATTGAGAATATGTTTTTCGTCTCAGCCAATCCCTGGGTGAGTTTCACCAGTTTTGATTTAAACGTGGCCAATATGGACAA
CTTCTTCGCCCCCGTTTTCACCATGGGCAAATATTATACGCAAGGCGACAAGGTGCTGATGCCGCTGGCGATTCAGGTTCATCATGCCGTTTGT
GATGGCTTCCATGTCGGCAGAATGCTTAATGAATTACAACAGTACTGCGATGAGTGGCAGGCGGGGCGTAATCTAGAGGATCCGGCTTACTAAA
AGCCAGATAACAGTATGCGTATTTGCGCGCTGATTTTTGCGGTATAAGAATATATACTGATATGTATACCCGAAGTATGTCAAAAAGAGGTATG
CTATGAAGCAGCGTATTACAGTGACAGTTGACAGCGACAGCTATCAGTTGCTCAAGGCATATATGATGTCAATATCTCCGGTCTGGTAAGCACA
ACCATGCAGAATGAAGCCCGTCGTCTGCGTGCCGAACGCTGGAAAGCGGAAAATCAGGAAGGGATGGCTGAGGTCGCCCGGTTTATTGAAATGA
ACGGCTCTTTTGCTGACGAGAACAGGGGCTGGTGAAATGCAGTTTAAGGTTTACACCTATAAAAGAGAGAGCCGTTATCGTCTGTTTGTGGATG
TACAGAGTGATATTATTGACACGCCCGGGCGACGGATGGTGATCCCCCTGGCCAGTGCACGTCTGCTGTCAGATAAAGTCCCCCGTGAACTTTA
CCCGGTGGTGCATATCGGGGATGAAAGCTGGCGCATGATGACCACCGATATGGCCAGTGTGCCGGTCTCCGTTATCGGGGAAGAAGTGGCTGAT
CTCAGCCACCGCGAAAATGACATCAAAAACGCCATTAACCTGATGTTCTGGGGAATATAAATGTCAGGCTCCCTTATACACAGCCAGTCTGCAG
GTCGACCATAGTGACTGGATATGTTGTGTTTTACAGTATTATGTAGTCTGTTTTTTATGCAAAATCTAATTTAATATATTGATATTTATATCAT
TTTACGTTTCTCGTTCAGCTTTCTTGTACAAAGTGGTGACGTAAGCTAGTTTAAACCCGCTGATCAGCCTCGACTGTGCCTTCTAAGATCCAGA
CATGATAAGATACATTGATGAGTTTGGACAAACCACAACTAGAATGCAGTGAAAAAAATGCTTTATTTGTGAAATTTGTGATGCTATTGCTTTA
TTTGTAACCATTATAAGCTGCAATAAACAAGTTCTCGGTACCCGATCCAGACATGATAAGATACATTGATGAGTTTGGACAAACCACAACTAGA
ATGCAGTGAAAAAAATGCTTTATTTGTGAAATTTGTGATGCTATTGCTTTATTTGTAACCATTATAAGCTGCAATAAACAAGTTCAAGCTAGCT
GGCCTCGAGGCTGATCTGCACCCAATCGGCAGGCACGGGCGGCGATCTCCAATCTGCGGGATCAGTCAGATCACCCGAGTGCGTGGGCATGACA
ATCGTGCCCTGGGGACCAACACAATCCAGAAGGGCCTGAATCACTGCGACCGGCCCTCCCGCGACCCAGCCGAGCGAGCTTAGCGAACTGTGGA
CGAGAACTGTGCCACCAAGCGTAAGGCCGTTCTCTCGCATTTGCCTTGCTAGGCTCGCGCGAGTTGCTGGCTGAGGCGTTCTCGAAATCAGCTC
TTGTTCGGTCGGCATCTACTCTATTCCTTTGCCCTCGGACGAGTGCTGGGGCGTCGGTTTCCACTATCGGCGAGTACTTCTACACAGCCATCGG
TCCAGACGGCCGCGCTTCTGCGGGCGATTTGTGTACGCCCGACAGTCCCGGCTCCGGATCGGACGATTGCGTCGCATCGACCCTGCGCCCAAGC
TGCATCATCGAAATTGCCGTCAACCAAGCTCTGATAGAGTTGGTCAAGACCAATGCGGAGCATATACGCCCGGAGCCGCGGCGATCCTGCAAGC
TCCGGATGCCTCCGCTCGAAGTAGCGCGTCTGCTGCTCCATACAAGCCAACCACGGCCTCCAGAAGAAGATGTTGGCGACCTCGTATTGGGAAT
CCCCGAACATCGCCTCGCTCCAGTCAATGACCGCTGTTATGCGGCCATTGTCCGTCAGGACATTGTTGGAGCCGAAATCCGCGTGCACGAGGTG
CCGGACTTCGGGGCAGTCCTCGGCCCAAAGCATCAGCTCATCGAGAGCCTGCGCGACGGACGCACTGACGGTGTCGTCCATCACAGTTTGCCAG
TGATACACATGGGGATCAGCAATCGCGCATATGAAATCACGCCATGTAGTGTATTGACCGATTCCTTGCGGTCCGAATGGGCCGAACCCGCTCG
TCTGGCTAAGATCGGCCGCAGCGATCGCATCCATGGCCTCCGCGACCGGCTGCAGAACAGCGGGCAGTTCGGTTTCAGGCAGGTCTTGCAACGT
GACACCCTGTGCACGGCGGGAGATGCAATAGGTCAGGCTCTCGCTGAATTCCCCAATGTCAAGCACTTCCGGAATCGGGAGCGCGGCCGATGCA
AAGTGCCGATAAACATAACGATCTTTGTAGAAACCATCGCCGCAGCTATTTACCCGCAGGACATATCCACGCCCTCCTACATCGAAGCTGAAAG
CACGAGATTCTTCGCCCTCCGAGAGCTGCATCAGGTCGGAGACGCTGTCGAACTTTTCGATCAGAAACTTCTCGACAGACGTCGCGGTACGCTC
AGGCTTTCCCATGGTGGCCAAGCTGGGGATCCCCATAACCTGTTGTAATTTATAATTTATATTTCCTTTCTTAATAAATAAATAAATAGTCAAG
TTTATGTTTGAGTTTTATGATTTATATTTTAAGTTATTTCAACTGCAACACCAGCACCACGACCTACTCACAGCAAAAAACGTACAAGAAGGAA
AGAAGGAATAAAAAGAGTGGTATTCTCTTACAATATGTTTTATGGCATAAAAGGTGTGGCCATTCATATCAAATATAAAGTAGTGTTGTTTAAC
GTTATTTTTGTAGGTTGAATAGTATATTCCAACAGATGATGAGGGGTTCCCAATCCTAAACCCATTTGCCGTTCCCAGAAGCATGAAACCACCA
CGCACCGGATCCTCTAGAGTCGACCTGCAGGCATGCAAGCTAACAACAACAATTGCATTCATTTTATGTTTCAGGTTCAGGGGGAGGTGTGGGA
GGTTTTTTAAAGCAAGTAAAACCTCTACAAATGTGGTATGGCTGATTATGATCAGTCGACCTGCAGGCATGCAAGCTTGGCGTAATCATGGTCA
TAGCTGTTTCCTGTGTGAAATTGTTATCCGCTCACAATTCCACACAACATACGAGCCGGAAGCATAAAGTGTAAAGCCTGGGGTGCCTAATGAG
TGAGCTAACTCACATTAATTGCGTTGCGCTCACTGCCCGCTTTCCAGTCGGGAAACCTGTCGTGCCAGCTGCATTAATGAATCGGCCAACGCGC
GGGGAGAGGCGGTTTGCGTATTGGGCGCTCTTCCGCTTCCTCGCTCACTGACTCGCTGCGCTCGGTCGTTCGGCTGCGGCGAGCGGTATCAGCT
CACTCAAAGGCGGTAATACGGTTATCCACAGAATCAGGGGATAACGCAGGAAAGAACATGTGAGCAAAAGGCCAGCAAAAGGCCAGGAACCGTA
AAAAGGCCGCGTTGCTGGCGTTTTTCCATAGGCTCCGCCCCCCTGACGAGCATCACAAAAATCGACGCTCAAGTCAGAGGTGGCGAAACCCGAC
AGGACTATAAAGATACCAGGCGTTTCCCCCTGGAAGCTCCCTCGTGCGCTCTCCTGTTCCGACCCTGCCGCTTACCGGATACCTGTCCGCCTTT
CTCCCTTCGGGAAGCGTGGCGCTTTCTCATAGCTCACGCTGTAGGTATCTCAGTTCGGTGTAGGTCGTTCGCTCCAAGCTGGGCTGTGTGCACG
AACCCCCCGTTCAGCCCGACCGCTGCGCCTTATCCGGTAACTATCGTCTTGAGTCCAACCCGGTAAGACACGACTTATCGCCACTGGCAGCAGC

CACTGGTAACAGGATTAGCAGAGCGAGGTATGTAGGCGGTGCTACAGAGTTCTTGAAGTGGTGGCCTAACTACGGCTACACTAGAAGGACAGTA
TTTGGTATCTGCGCTCTGCTGAAGCCAGTTACCTTCGGAAAAAGAGTTGGTAGCTCTTGATCCGGCAAACAAACCACCGCTGGTAGCGGTGGTT
TTTTTGTTTGCAAGCAGCAGATTACGCGCAGAAAAAAAGGATCTCAAGAAGATCCTTTGATCTTTTCTACGGGGTCTGACGCTCAGTGGAACGA
AAACTCACGTTAAGGGATTTTGGTCATGAGATTATCAAAAAGGATCTTCACCTAGATCCTTTTAAATTAAAAATGAAGTTTTAAATCAATCTAA
AGTATATATGAGTAAACTTGGTCTGACAGTTACCAATGCTTAATCAGTGAGGCACCTATCTCAGCGATCTGTCTATTTCGTTCATCCATAGTTG
CCTGACTCCCCGTCGTGTAGATAACTACGATACGGGAGGGCTTACCATCTGGCCCCAGTGCTGCAATGATACCGCGAGACCCACGCTCACCGGC
TCCAGATTTATCAGCAATAAACCAGCCAGCCGGAAGGGCCGAGCGCAGAAGTGGTCCTGCAACTTTATCCGCCTCCATCCAGTCTATTAATTGT
TGCCGGGAAGCTAGAGTAAGTAGTTCGCCAGTTAATAGTTTGCGCAACGTTGTTGCCATTGCTACAGGCATCGTGGTGTCACGCTCGTCGTTTG
GTATGGCTTCATTCAGCTCCGGTTCCCAACGATCAAGGCGAGTTACATGATCCCCCATGTTGTGCAAAAAAGCGGTTAGCTCCTTCGGTCCTCC
GATCGTTGTCAGAAGTAAGTTGGCCGCAGTGTTATCACTCATGGTTATGGCAGCACTGCATAATTCTCTTACTGTCATGCCATCCGTAAGATGC
TTTTCTGTGACTGGTGAGTACTCAACCAAGTCATTCTGAGAATAGTGTATGCGGCGACCGAGTTGCTCTTGCCCGGCGTCAATACGGGATAATA
CCGCGCCACATAGCAGAACTTTAAAAGTGCTCATCATTGGAAAACGTTCTTCGGGGCGAAAACTCTCAAGGATCTTACCGCTGTTGAGATCCAG
TTCGATGTAACCCACTCGTGCACCCAACTGATCTTCAGCATCTTTTACTTTCACCAGCGTTTCTGGGTGAGCAAAAACAGGAAGGCAAAATGCC
GCAAAAAAGGGAATAAGGGCGACACGGAAATGTTGAATACTCATACTCTTCCTTTTTCAATATTATTGAAGCATTTATCAGGGTTATTGTCTCA
TGAGCGGATACATATTTGAATGTATTTAGAAAAATAAACAAATAGGGGTTCCGCGCACATTTCCCCGAAAAGTGCCACCTGACGTCTAAGAAAC
CATTATTATCATGACATTAACCTATAAAAATAGGCGTATCACGAGGCCCTTTCGT

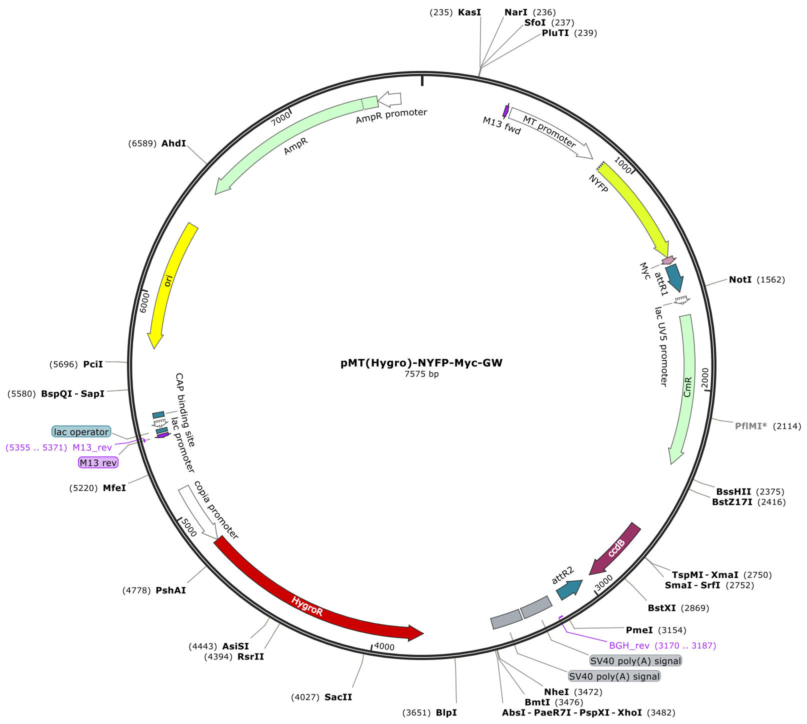

> pMT(Hygro)-CYFP-HA-GW

TCGCGCGTTTCGGTGATGACGGTGAAAACCTCTGACACATGCAGCTCCCGGAGACGGTCACAGCTTGTCTGTAAGCGGATGCCGGGAGCAGACA
AGCCCGTCAGGGCGCGTCAGCGGGTGTTGGCGGGTGTCGGGGCTGGCTTAACTATGCGGCATCAGAGCAGATTGTACTGAGAGTGCACCATATG
CGGTGTGAAATACCGCACAGATGCGTAAGGAGAAAATACCGCATCAGGCGCCATTCGCCATTCAGGCTGCGCAACTGTTGGGAAGGGCGATCGG
TGCGGGCCTCTTCGCTATTACGCCAGCTGGCGAAAGGGGGATGTGCTGCAAGGCGATTAAGTTGGGTAACGCCAGGGTTTTCCCAGTCACGACG
TTGTAAAACGACGGCCAGTGCCAGTGAATTAATTCGTTGCAGGACAGGATGTGGTGCCCGATGTGACTAGCTCTTTGCTGCAGGCCGTCCTATC
CTCTGGTTCCGATAAGAGACCCAGAACTCCGGCCCCCCACCGCCCACCGCCACCCCCATACATATGTGGTACGCAAGTAAGAGTGCCTGCGCAT
GCCCCATGTGCCCCACCAAGAGTTTTGCATCCCATACAAGTCCCCAAAGTGGAGAACCGAACCAATTCTTCGCGGGCAGAACAAAAGCTTCTGC
ACACGTCTCCACTCGAATTTGGAGCCGGCCGGCGTGTGCAAAAGAGGTGAATCGAACGAAAGACCCGTGTGTAAAGCCGCGTTTCCAAAATGTA
TAAAACCGAGAGCATCTGGCCAATGTGCATCAGTTGTGGTCAGCAGCAAAATCAAGTGAATCATCTCAGTGCAACTAAAGGGGGGATCTAGATC
GGGGTACCACTGCAGTGAATTCGGAGaattcATGGACAAGCAGAAGAACGGCATCAAGGTGAACTTCAAGATCCGCCACAACATCGAGGACGGC
AGCGTGCAGCTCGCCGACCACTACCAGCAGAACACCCCCATCGGCGACGGCCCCGTGCTGCTGCCCGACAACCACTACCTGAGCTACCAGTCCA
AGCTGAGCAAAGACCCCAACGAGAAGCGCGATCACATGGTCCTGCTGGAGTTCGTGACCGCCGCCGGGATCACTCTCGGCATGGACGAGCTGTA
CAAGTACCCATACGATGTTCCAGATTACGCTtctagATCAACAAGTTTGTACAAAAAAGCTGAACGAGAAACGTAAAATGATATAAATATCAAT
ATATTAAATTAGATTTTGCATAAAAAACAGACTACATAATACTGTAAAACACAACATATCCAGTCACTATGGCGGCCGCATTAGGCACCCCAGG
CTTTACACTTTATGCTTCCGGCTCGTATAATGTGTGGATTTTGAGTTAGGATCCGTCGAGATTTTCAGGAGCTAAGGAAGCTAAAATGGAGAAA
AAAATCACTGGATATACCACCGTTGATATATCCCAATGGCATCGTAAAGAACATTTTGAGGCATTTCAGTCAGTTGCTCAATGTACCTATAACC

AGACCGTTCAGCTGGATATTACGGCCTTTTTAAAGACCGTAAAGAAAAATAAGCACAAGTTTTATCCGGCCTTTATTCACATTCTTGCCCGCCT
GATGAATGCTCATCCGGAATTCCGTATGGCAATGAAAGACGGTGAGCTGGTGATATGGGATAGTGTTCACCCTTGTTACACCGTTTTCCATGAG
CAAACTGAAACGTTTTCATCGCTCTGGAGTGAATACCACGACGATTTCCGGCAGTTTCTACACATATATTCGCAAGATGTGGCGTGTTACGGTG
AAAACCTGGCCTATTTCCCTAAAGGGTTTATTGAGAATATGTTTTTCGTCTCAGCCAATCCCTGGGTGAGTTTCACCAGTTTTGATTTAAACGT
GGCCAATATGGACAACTTCTTCGCCCCCGTTTTCACCATGGGCAAATATTATACGCAAGGCGACAAGGTGCTGATGCCGCTGGCGATTCAGGTT
CATCATGCCGTTTGTGATGGCTTCCATGTCGGCAGAATGCTTAATGAATTACAACAGTACTGCGATGAGTGGCAGGCGGGGCGTAATCTAGAGG
ATCCGGCTTACTAAAAGCCAGATAACAGTATGCGTATTTGCGCGCTGATTTTTGCGGTATAAGAATATATACTGATATGTATACCCGAAGTATG
TCAAAAAGAGGTATGCTATGAAGCAGCGTATTACAGTGACAGTTGACAGCGACAGCTATCAGTTGCTCAAGGCATATATGATGTCAATATCTCC
GGTCTGGTAAGCACAACCATGCAGAATGAAGCCCGTCGTCTGCGTGCCGAACGCTGGAAAGCGGAAAATCAGGAAGGGATGGCTGAGGTCGCCC
GGTTTATTGAAATGAACGGCTCTTTTGCTGACGAGAACAGGGGCTGGTGAAATGCAGTTTAAGGTTTACACCTATAAAAGAGAGAGCCGTTATC
GTCTGTTTGTGGATGTACAGAGTGATATTATTGACACGCCCGGGCGACGGATGGTGATCCCCCTGGCCAGTGCACGTCTGCTGTCAGATAAAGT
CCCCCGTGAACTTTACCCGGTGGTGCATATCGGGGATGAAAGCTGGCGCATGATGACCACCGATATGGCCAGTGTGCCGGTCTCCGTTATCGGG
GAAGAAGTGGCTGATCTCAGCCACCGCGAAAATGACATCAAAAACGCCATTAACCTGATGTTCTGGGGAATATAAATGTCAGGCTCCCTTATAC
ACAGCCAGTCTGCAGGTCGACCATAGTGACTGGATATGTTGTGTTTTACAGTATTATGTAGTCTGTTTTTTATGCAAAATCTAATTTAATATAT
TGATATTTATATCATTTTACGTTTCTCGTTCAGCTTTCTTGTACAAAGTGGTGACGTAAGCTAGTTTAAACCCGCTGATCAGCCTCGACTGTGC
CTTCTAAGATCCAGACATGATAAGATACATTGATGAGTTTGGACAAACCACAACTAGAATGCAGTGAAAAAAATGCTTTATTTGTGAAATTTGT
GATGCTATTGCTTTATTTGTAACCATTATAAGCTGCAATAAACAAGTTCTCGGTACCCGATCCAGACATGATAAGATACATTGATGAGTTTGGA
CAAACCACAACTAGAATGCAGTGAAAAAAATGCTTTATTTGTGAAATTTGTGATGCTATTGCTTTATTTGTAACCATTATAAGCTGCAATAAAC
AAGTTCAAGCTAGCTGGCCTCGAGGCTGATCTGCACCCAATCGGCAGGCACGGGCGGCGATCTCCAATCTGCGGGATCAGTCAGATCACCCGAG
TGCGTGGGCATGACAATCGTGCCCTGGGGACCAACACAATCCAGAAGGGCCTGAATCACTGCGACCGGCCCTCCCGCGACCCAGCCGAGCGAGC
TTAGCGAACTGTGGACGAGAACTGTGCCACCAAGCGTAAGGCCGTTCTCTCGCATTTGCCTTGCTAGGCTCGCGCGAGTTGCTGGCTGAGGCGT
TCTCGAAATCAGCTCTTGTTCGGTCGGCATCTACTCTATTCCTTTGCCCTCGGACGAGTGCTGGGGCGTCGGTTTCCACTATCGGCGAGTACTT
CTACACAGCCATCGGTCCAGACGGCCGCGCTTCTGCGGGCGATTTGTGTACGCCCGACAGTCCCGGCTCCGGATCGGACGATTGCGTCGCATCG
ACCCTGCGCCCAAGCTGCATCATCGAAATTGCCGTCAACCAAGCTCTGATAGAGTTGGTCAAGACCAATGCGGAGCATATACGCCCGGAGCCGC
GGCGATCCTGCAAGCTCCGGATGCCTCCGCTCGAAGTAGCGCGTCTGCTGCTCCATACAAGCCAACCACGGCCTCCAGAAGAAGATGTTGGCGA
CCTCGTATTGGGAATCCCCGAACATCGCCTCGCTCCAGTCAATGACCGCTGTTATGCGGCCATTGTCCGTCAGGACATTGTTGGAGCCGAAATC
CGCGTGCACGAGGTGCCGGACTTCGGGGCAGTCCTCGGCCCAAAGCATCAGCTCATCGAGAGCCTGCGCGACGGACGCACTGACGGTGTCGTCC
ATCACAGTTTGCCAGTGATACACATGGGGATCAGCAATCGCGCATATGAAATCACGCCATGTAGTGTATTGACCGATTCCTTGCGGTCCGAATG
GGCCGAACCCGCTCGTCTGGCTAAGATCGGCCGCAGCGATCGCATCCATGGCCTCCGCGACCGGCTGCAGAACAGCGGGCAGTTCGGTTTCAGG
CAGGTCTTGCAACGTGACACCCTGTGCACGGCGGGAGATGCAATAGGTCAGGCTCTCGCTGAATTCCCCAATGTCAAGCACTTCCGGAATCGGG
AGCGCGGCCGATGCAAAGTGCCGATAAACATAACGATCTTTGTAGAAACCATCGCCGCAGCTATTTACCCGCAGGACATATCCACGCCCTCCTA
CATCGAAGCTGAAAGCACGAGATTCTTCGCCCTCCGAGAGCTGCATCAGGTCGGAGACGCTGTCGAACTTTTCGATCAGAAACTTCTCGACAGA
CGTCGCGGTACGCTCAGGCTTTCCCATGGTGGCCAAGCTGGGGATCCCCATAACCTGTTGTAATTTATAATTTATATTTCCTTTCTTAATAAAT
AAATAAATAGTCAAGTTTATGTTTGAGTTTTATGATTTATATTTTAAGTTATTTCAACTGCAACACCAGCACCACGACCTACTCACAGCAAAAA
ACGTACAAGAAGGAAAGAAGGAATAAAAAGAGTGGTATTCTCTTACAATATGTTTTATGGCATAAAAGGTGTGGCCATTCATATCAAATATAAA
GTAGTGTTGTTTAACGTTATTTTTGTAGGTTGAATAGTATATTCCAACAGATGATGAGGGGTTCCCAATCCTAAACCCATTTGCCGTTCCCAGA
AGCATGAAACCACCACGCACCGGATCCTCTAGAGTCGACCTGCAGGCATGCAAGCTAACAACAACAATTGCATTCATTTTATGTTTCAGGTTCA
GGGGGAGGTGTGGGAGGTTTTTTAAAGCAAGTAAAACCTCTACAAATGTGGTATGGCTGATTATGATCAGTCGACCTGCAGGCATGCAAGCTTG
GCGTAATCATGGTCATAGCTGTTTCCTGTGTGAAATTGTTATCCGCTCACAATTCCACACAACATACGAGCCGGAAGCATAAAGTGTAAAGCCT
GGGGTGCCTAATGAGTGAGCTAACTCACATTAATTGCGTTGCGCTCACTGCCCGCTTTCCAGTCGGGAAACCTGTCGTGCCAGCTGCATTAATG
AATCGGCCAACGCGCGGGGAGAGGCGGTTTGCGTATTGGGCGCTCTTCCGCTTCCTCGCTCACTGACTCGCTGCGCTCGGTCGTTCGGCTGCGG
CGAGCGGTATCAGCTCACTCAAAGGCGGTAATACGGTTATCCACAGAATCAGGGGATAACGCAGGAAAGAACATGTGAGCAAAAGGCCAGCAAA
AGGCCAGGAACCGTAAAAAGGCCGCGTTGCTGGCGTTTTTCCATAGGCTCCGCCCCCCTGACGAGCATCACAAAAATCGACGCTCAAGTCAGAG
GTGGCGAAACCCGACAGGACTATAAAGATACCAGGCGTTTCCCCCTGGAAGCTCCCTCGTGCGCTCTCCTGTTCCGACCCTGCCGCTTACCGGA
TACCTGTCCGCCTTTCTCCCTTCGGGAAGCGTGGCGCTTTCTCATAGCTCACGCTGTAGGTATCTCAGTTCGGTGTAGGTCGTTCGCTCCAAGC
TGGGCTGTGTGCACGAACCCCCCGTTCAGCCCGACCGCTGCGCCTTATCCGGTAACTATCGTCTTGAGTCCAACCCGGTAAGACACGACTTATC
GCCACTGGCAGCAGCCACTGGTAACAGGATTAGCAGAGCGAGGTATGTAGGCGGTGCTACAGAGTTCTTGAAGTGGTGGCCTAACTACGGCTAC
ACTAGAAGGACAGTATTTGGTATCTGCGCTCTGCTGAAGCCAGTTACCTTCGGAAAAAGAGTTGGTAGCTCTTGATCCGGCAAACAAACCACCG
CTGGTAGCGGTGGTTTTTTTGTTTGCAAGCAGCAGATTACGCGCAGAAAAAAAGGATCTCAAGAAGATCCTTTGATCTTTTCTACGGGGTCTGA
CGCTCAGTGGAACGAAAACTCACGTTAAGGGATTTTGGTCATGAGATTATCAAAAAGGATCTTCACCTAGATCCTTTTAAATTAAAAATGAAGT
TTTAAATCAATCTAAAGTATATATGAGTAAACTTGGTCTGACAGTTACCAATGCTTAATCAGTGAGGCACCTATCTCAGCGATCTGTCTATTTC
GTTCATCCATAGTTGCCTGACTCCCCGTCGTGTAGATAACTACGATACGGGAGGGCTTACCATCTGGCCCCAGTGCTGCAATGATACCGCGAGA
CCCACGCTCACCGGCTCCAGATTTATCAGCAATAAACCAGCCAGCCGGAAGGGCCGAGCGCAGAAGTGGTCCTGCAACTTTATCCGCCTCCATC
CAGTCTATTAATTGTTGCCGGGAAGCTAGAGTAAGTAGTTCGCCAGTTAATAGTTTGCGCAACGTTGTTGCCATTGCTACAGGCATCGTGGTGT
CACGCTCGTCGTTTGGTATGGCTTCATTCAGCTCCGGTTCCCAACGATCAAGGCGAGTTACATGATCCCCCATGTTGTGCAAAAAAGCGGTTAG
CTCCTTCGGTCCTCCGATCGTTGTCAGAAGTAAGTTGGCCGCAGTGTTATCACTCATGGTTATGGCAGCACTGCATAATTCTCTTACTGTCATG
CCATCCGTAAGATGCTTTTCTGTGACTGGTGAGTACTCAACCAAGTCATTCTGAGAATAGTGTATGCGGCGACCGAGTTGCTCTTGCCCGGCGT
CAATACGGGATAATACCGCGCCACATAGCAGAACTTTAAAAGTGCTCATCATTGGAAAACGTTCTTCGGGGCGAAAACTCTCAAGGATCTTACC
GCTGTTGAGATCCAGTTCGATGTAACCCACTCGTGCACCCAACTGATCTTCAGCATCTTTTACTTTCACCAGCGTTTCTGGGTGAGCAAAAACA
GGAAGGCAAAATGCCGCAAAAAAGGGAATAAGGGCGACACGGAAATGTTGAATACTCATACTCTTCCTTTTTCAATATTATTGAAGCATTTATC
AGGGTTATTGTCTCATGAGCGGATACATATTTGAATGTATTTAGAAAAATAAACAAATAGGGGTTCCGCGCACATTTCCCCGAAAAGTGCCACC
TGACGTCTAAGAAACCATTATTATCATGACATTAACCTATAAAAATAGGCGTATCACGAGGCCCTTTCGT

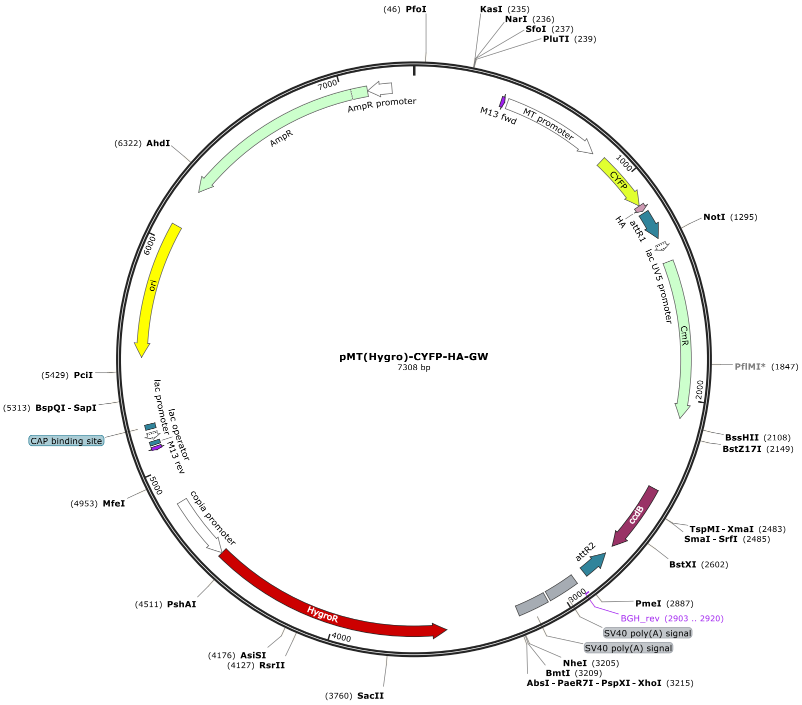

>pMT(Hygro)-mCherry-GW

TCGCGCGTTTCGGTGATGACGGTGAAAACCTCTGACACATGCAGCTCCCGGAGACGGTCACAGCTTGTCTGTAAGCGGATGCCGGGAGCAGACA
AGCCCGTCAGGGCGCGTCAGCGGGTGTTGGCGGGTGTCGGGGCTGGCTTAACTATGCGGCATCAGAGCAGATTGTACTGAGAGTGCACCATATG
CGGTGTGAAATACCGCACAGATGCGTAAGGAGAAAATACCGCATCAGGCGCCATTCGCCATTCAGGCTGCGCAACTGTTGGGAAGGGCGATCGG
TGCGGGCCTCTTCGCTATTACGCCAGCTGGCGAAAGGGGGATGTGCTGCAAGGCGATTAAGTTGGGTAACGCCAGGGTTTTCCCAGTCACGACG
TTGTAAAACGACGGCCAGTGCCAGTGAATTAATTCGTTGCAGGACAGGATGTGGTGCCCGATGTGACTAGCTCTTTGCTGCAGGCCGTCCTATC
CTCTGGTTCCGATAAGAGACCCAGAACTCCGGCCCCCCACCGCCCACCGCCACCCCCATACATATGTGGTACGCAAGTAAGAGTGCCTGCGCAT
GCCCCATGTGCCCCACCAAGAGTTTTGCATCCCATACAAGTCCCCAAAGTGGAGAACCGAACCAATTCTTCGCGGGCAGAACAAAAGCTTCTGC
ACACGTCTCCACTCGAATTTGGAGCCGGCCGGCGTGTGCAAAAGAGGTGAATCGAACGAAAGACCCGTGTGTAAAGCCGCGTTTCCAAAATGTA
TAAAACCGAGAGCATCTGGCCAATGTGCATCAGTTGTGGTCAGCAGCAAAATCAAGTGAATCATCTCAGTGCAACTAAAGGGGGGATCTAGATC
GGGGTACCACTGCAGTGAATTCGGAGCTCACCATGGTGAGCAAGGGCGAGGAGGATAACATGGCCATCATCAAGGAGTTCATGCGCTTCAAGGT
GCACATGGAGGGCTCCGTGAACGGCCACGAGTTCGAGATCGAGGGCGAGGGCGAGGGCCGCCCCTACGAGGGCACCCAGACCGCCAAGCTGAAG
GTGACCAAGGGTGGCCCCCTGCCCTTCGCCTGGGACATCCTGTCCCCTCAGTTCATGTACGGCTCCAAGGCCTACGTGAAGCACCCCGCCGACA
TCCCCGACTACTTGAAGCTGTCCTTCCCCGAGGGCTTCAAGTGGGAGCGCGTGATGAACTTCGAGGACGGCGGCGTGGTGACCGTGACCCAGGA
CTCCTCCCTGCAGGACGGCGAGTTCATCTACAAGGTGAAGCTGCGCGGCACCAACTTCCCCTCCGACGGCCCCGTAATGCAGAAGAAGACCATG
GGCTGGGAGGCCTCCTCCGAGCGGATGTACCCCGAGGACGGCGCCCTGAAGGGCGAGATCAAGCAGAGGCTGAAGCTGAAGGACGGCGGCCACT
ACGACGCTGAGGTCAAGACCACCTACAAGGCCAAGAAGCCCGTGCAGCTGCCCGGCGCCTACAACGTCAACATCAAGTTGGACATCACCTCCCA
CAACGAGGACTACACCATCGTGGAACAGTACGAACGCGCCGAGGGCCGCCACTCCACCGGCGGCATGGACGAGCTGTACAAGCACCGGTATACA
AGTTTGTACAAAAAAGCTGAACGAGAAACGTAAAATGATATAAATATCAATATATTAAATTAGATTTTGCATAAAAAACAGACTACATAATACT
GTAAAACACAACATATCCAGTCACTATGGCGGCCGCATTAGGCACCCCAGGCTTTACACTTTATGCTTCCGGCTCGTATAATGTGTGGATTTTG
AGTTAGGATCCGTCGAGATTTTCAGGAGCTAAGGAAGCTAAAATGGAGAAAAAAATCACTGGATATACCACCGTTGATATATCCCAATGGCATC
GTAAAGAACATTTTGAGGCATTTCAGTCAGTTGCTCAATGTACCTATAACCAGACCGTTCAGCTGGATATTACGGCCTTTTTAAAGACCGTAAA
GAAAAATAAGCACAAGTTTTATCCGGCCTTTATTCACATTCTTGCCCGCCTGATGAATGCTCATCCGGAATTCCGTATGGCAATGAAAGACGGT
GAGCTGGTGATATGGGATAGTGTTCACCCTTGTTACACCGTTTTCCATGAGCAAACTGAAACGTTTTCATCGCTCTGGAGTGAATACCACGACG
ATTTCCGGCAGTTTCTACACATATATTCGCAAGATGTGGCGTGTTACGGTGAAAACCTGGCCTATTTCCCTAAAGGGTTTATTGAGAATATGTT
TTTCGTCTCAGCCAATCCCTGGGTGAGTTTCACCAGTTTTGATTTAAACGTGGCCAATATGGACAACTTCTTCGCCCCCGTTTTCACCATGGGC
AAATATTATACGCAAGGCGACAAGGTGCTGATGCCGCTGGCGATTCAGGTTCATCATGCCGTTTGTGATGGCTTCCATGTCGGCAGAATGCTTA
ATGAATTACAACAGTACTGCGATGAGTGGCAGGCGGGGCGTAATCTAGAGGATCCGGCTTACTAAAAGCCAGATAACAGTATGCGTATTTGCGC
GCTGATTTTTGCGGTATAAGAATATATACTGATATGTATACCCGAAGTATGTCAAAAAGAGGTATGCTATGAAGCAGCGTATTACAGTGACAGT
TGACAGCGACAGCTATCAGTTGCTCAAGGCATATATGATGTCAATATCTCCGGTCTGGTAAGCACAACCATGCAGAATGAAGCCCGTCGTCTGC
GTGCCGAACGCTGGAAAGCGGAAAATCAGGAAGGGATGGCTGAGGTCGCCCGGTTTATTGAAATGAACGGCTCTTTTGCTGACGAGAACAGGGG
CTGGTGAAATGCAGTTTAAGGTTTACACCTATAAAAGAGAGAGCCGTTATCGTCTGTTTGTGGATGTACAGAGTGATATTATTGACACGCCCGG
GCGACGGATGGTGATCCCCCTGGCCAGTGCACGTCTGCTGTCAGATAAAGTCCCCCGTGAACTTTACCCGGTGGTGCATATCGGGGATGAAAGC

TGGCGCATGATGACCACCGATATGGCCAGTGTGCCGGTCTCCGTTATCGGGGAAGAAGTGGCTGATCTCAGCCACCGCGAAAATGACATCAAAA
ACGCCATTAACCTGATGTTCTGGGGAATATAAATGTCAGGCTCCCTTATACACAGCCAGTCTGCAGGTCGACCATAGTGACTGGATATGTTGTG
TTTTACAGTATTATGTAGTCTGTTTTTTATGCAAAATCTAATTTAATATATTGATATTTATATCATTTTACGTTTCTCGTTCAGCTTTCTTGTA
CAAAGTGGTGACGTAAGCTAGTTTAAACCCGCTGATCAGCCTCGACTGTGCCTTCTAAGATCCAGACATGATAAGATACATTGATGAGTTTGGA
CAAACCACAACTAGAATGCAGTGAAAAAAATGCTTTATTTGTGAAATTTGTGATGCTATTGCTTTATTTGTAACCATTATAAGCTGCAATAAAC
AAGTTCTCGGTACCCGATCCAGACATGATAAGATACATTGATGAGTTTGGACAAACCACAACTAGAATGCAGTGAAAAAAATGCTTTATTTGTG
AAATTTGTGATGCTATTGCTTTATTTGTAACCATTATAAGCTGCAATAAACAAGTTCAAGCTAGCTGGCCTCGAGGCTGATCTGCACCCAATCG
GCAGGCACGGGCGGCGATCTCCAATCTGCGGGATCAGTCAGATCACCCGAGTGCGTGGGCATGACAATCGTGCCCTGGGGACCAACACAATCCA
GAAGGGCCTGAATCACTGCGACCGGCCCTCCCGCGACCCAGCCGAGCGAGCTTAGCGAACTGTGGACGAGAACTGTGCCACCAAGCGTAAGGCC
GTTCTCTCGCATTTGCCTTGCTAGGCTCGCGCGAGTTGCTGGCTGAGGCGTTCTCGAAATCAGCTCTTGTTCGGTCGGCATCTACTCTATTCCT
TTGCCCTCGGACGAGTGCTGGGGCGTCGGTTTCCACTATCGGCGAGTACTTCTACACAGCCATCGGTCCAGACGGCCGCGCTTCTGCGGGCGAT
TTGTGTACGCCCGACAGTCCCGGCTCCGGATCGGACGATTGCGTCGCATCGACCCTGCGCCCAAGCTGCATCATCGAAATTGCCGTCAACCAAG
CTCTGATAGAGTTGGTCAAGACCAATGCGGAGCATATACGCCCGGAGCCGCGGCGATCCTGCAAGCTCCGGATGCCTCCGCTCGAAGTAGCGCG
TCTGCTGCTCCATACAAGCCAACCACGGCCTCCAGAAGAAGATGTTGGCGACCTCGTATTGGGAATCCCCGAACATCGCCTCGCTCCAGTCAAT
GACCGCTGTTATGCGGCCATTGTCCGTCAGGACATTGTTGGAGCCGAAATCCGCGTGCACGAGGTGCCGGACTTCGGGGCAGTCCTCGGCCCAA
AGCATCAGCTCATCGAGAGCCTGCGCGACGGACGCACTGACGGTGTCGTCCATCACAGTTTGCCAGTGATACACATGGGGATCAGCAATCGCGC
ATATGAAATCACGCCATGTAGTGTATTGACCGATTCCTTGCGGTCCGAATGGGCCGAACCCGCTCGTCTGGCTAAGATCGGCCGCAGCGATCGC
ATCCATGGCCTCCGCGACCGGCTGCAGAACAGCGGGCAGTTCGGTTTCAGGCAGGTCTTGCAACGTGACACCCTGTGCACGGCGGGAGATGCAA
TAGGTCAGGCTCTCGCTGAATTCCCCAATGTCAAGCACTTCCGGAATCGGGAGCGCGGCCGATGCAAAGTGCCGATAAACATAACGATCTTTGT
AGAAACCATCGCCGCAGCTATTTACCCGCAGGACATATCCACGCCCTCCTACATCGAAGCTGAAAGCACGAGATTCTTCGCCCTCCGAGAGCTG
CATCAGGTCGGAGACGCTGTCGAACTTTTCGATCAGAAACTTCTCGACAGACGTCGCGGTACGCTCAGGCTTTCCCATGGTGGCCAAGCTGGGG
ATCCCCATAACCTGTTGTAATTTATAATTTATATTTCCTTTCTTAATAAATAAATAAATAGTCAAGTTTATGTTTGAGTTTTATGATTTATATT
TTAAGTTATTTCAACTGCAACACCAGCACCACGACCTACTCACAGCAAAAAACGTACAAGAAGGAAAGAAGGAATAAAAAGAGTGGTATTCTCT
TACAATATGTTTTATGGCATAAAAGGTGTGGCCATTCATATCAAATATAAAGTAGTGTTGTTTAACGTTATTTTTGTAGGTTGAATAGTATATT
CCAACAGATGATGAGGGGTTCCCAATCCTAAACCCATTTGCCGTTCCCAGAAGCATGAAACCACCACGCACCGGATCCTCTAGAGTCGACCTGC
AGGCATGCAAGCTAACAACAACAATTGCATTCATTTTATGTTTCAGGTTCAGGGGGAGGTGTGGGAGGTTTTTTAAAGCAAGTAAAACCTCTAC
AAATGTGGTATGGCTGATTATGATCAGTCGACCTGCAGGCATGCAAGCTTGGCGTAATCATGGTCATAGCTGTTTCCTGTGTGAAATTGTTATC
CGCTCACAATTCCACACAACATACGAGCCGGAAGCATAAAGTGTAAAGCCTGGGGTGCCTAATGAGTGAGCTAACTCACATTAATTGCGTTGCG
CTCACTGCCCGCTTTCCAGTCGGGAAACCTGTCGTGCCAGCTGCATTAATGAATCGGCCAACGCGCGGGGAGAGGCGGTTTGCGTATTGGGCGC
TCTTCCGCTTCCTCGCTCACTGACTCGCTGCGCTCGGTCGTTCGGCTGCGGCGAGCGGTATCAGCTCACTCAAAGGCGGTAATACGGTTATCCA
CAGAATCAGGGGATAACGCAGGAAAGAACATGTGAGCAAAAGGCCAGCAAAAGGCCAGGAACCGTAAAAAGGCCGCGTTGCTGGCGTTTTTCCA
TAGGCTCCGCCCCCCTGACGAGCATCACAAAAATCGACGCTCAAGTCAGAGGTGGCGAAACCCGACAGGACTATAAAGATACCAGGCGTTTCCC
CCTGGAAGCTCCCTCGTGCGCTCTCCTGTTCCGACCCTGCCGCTTACCGGATACCTGTCCGCCTTTCTCCCTTCGGGAAGCGTGGCGCTTTCTC
ATAGCTCACGCTGTAGGTATCTCAGTTCGGTGTAGGTCGTTCGCTCCAAGCTGGGCTGTGTGCACGAACCCCCCGTTCAGCCCGACCGCTGCGC
CTTATCCGGTAACTATCGTCTTGAGTCCAACCCGGTAAGACACGACTTATCGCCACTGGCAGCAGCCACTGGTAACAGGATTAGCAGAGCGAGG
TATGTAGGCGGTGCTACAGAGTTCTTGAAGTGGTGGCCTAACTACGGCTACACTAGAAGGACAGTATTTGGTATCTGCGCTCTGCTGAAGCCAG
TTACCTTCGGAAAAAGAGTTGGTAGCTCTTGATCCGGCAAACAAACCACCGCTGGTAGCGGTGGTTTTTTTGTTTGCAAGCAGCAGATTACGCG
CAGAAAAAAAGGATCTCAAGAAGATCCTTTGATCTTTTCTACGGGGTCTGACGCTCAGTGGAACGAAAACTCACGTTAAGGGATTTTGGTCATG
AGATTATCAAAAAGGATCTTCACCTAGATCCTTTTAAATTAAAAATGAAGTTTTAAATCAATCTAAAGTATATATGAGTAAACTTGGTCTGACA
GTTACCAATGCTTAATCAGTGAGGCACCTATCTCAGCGATCTGTCTATTTCGTTCATCCATAGTTGCCTGACTCCCCGTCGTGTAGATAACTAC
GATACGGGAGGGCTTACCATCTGGCCCCAGTGCTGCAATGATACCGCGAGACCCACGCTCACCGGCTCCAGATTTATCAGCAATAAACCAGCCA
GCCGGAAGGGCCGAGCGCAGAAGTGGTCCTGCAACTTTATCCGCCTCCATCCAGTCTATTAATTGTTGCCGGGAAGCTAGAGTAAGTAGTTCGC
CAGTTAATAGTTTGCGCAACGTTGTTGCCATTGCTACAGGCATCGTGGTGTCACGCTCGTCGTTTGGTATGGCTTCATTCAGCTCCGGTTCCCA
ACGATCAAGGCGAGTTACATGATCCCCCATGTTGTGCAAAAAAGCGGTTAGCTCCTTCGGTCCTCCGATCGTTGTCAGAAGTAAGTTGGCCGCA
GTGTTATCACTCATGGTTATGGCAGCACTGCATAATTCTCTTACTGTCATGCCATCCGTAAGATGCTTTTCTGTGACTGGTGAGTACTCAACCA
AGTCATTCTGAGAATAGTGTATGCGGCGACCGAGTTGCTCTTGCCCGGCGTCAATACGGGATAATACCGCGCCACATAGCAGAACTTTAAAAGT
GCTCATCATTGGAAAACGTTCTTCGGGGCGAAAACTCTCAAGGATCTTACCGCTGTTGAGATCCAGTTCGATGTAACCCACTCGTGCACCCAAC
TGATCTTCAGCATCTTTTACTTTCACCAGCGTTTCTGGGTGAGCAAAAACAGGAAGGCAAAATGCCGCAAAAAAGGGAATAAGGGCGACACGGA
AATGTTGAATACTCATACTCTTCCTTTTTCAATATTATTGAAGCATTTATCAGGGTTATTGTCTCATGAGCGGATACATATTTGAATGTATTTA
GAAAAATAAACAAATAGGGGTTCCGCGCACATTTCCCCGAAAAGTGCCACCTGACGTCTAAGAAACCATTATTATCATGACATTAACCTATAAA
AATAGGCGTATCACGAGGCCCTTTCGT

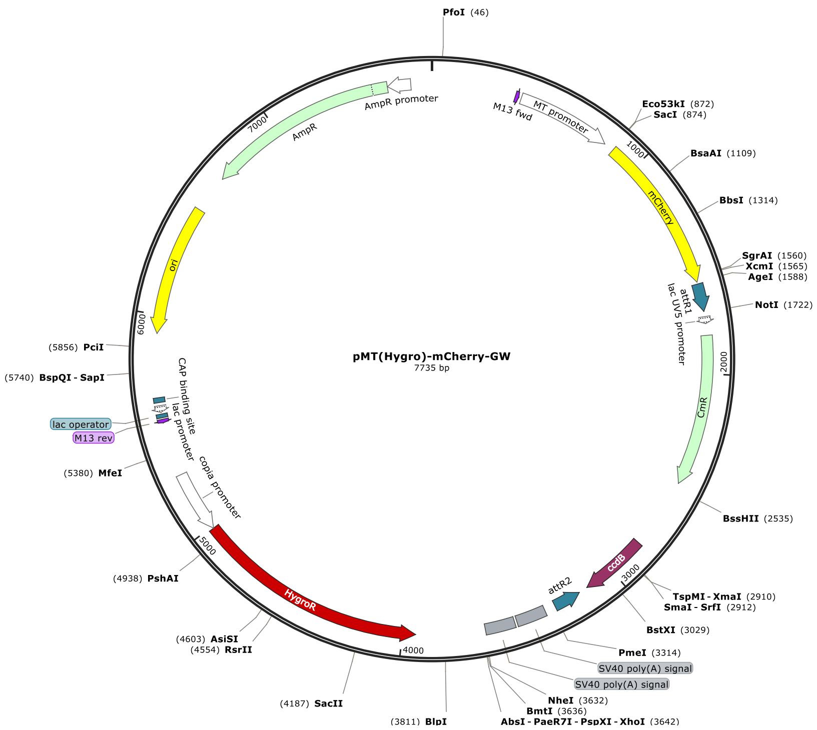
PfoI (46)
Eco53kI (872)
SacI (874)
BsaAI (1109)
BbsI (1314)
SgrAI (1560)
XcmI (1565)
AgeI (1588)
NotI (1722)
BssHII (2535)
TspMI - XmaI (2910)
SmaI - SrfI (2912)
BstXI (3029)
PmeI (3314)
SV40 poly(A) signal
SV40 poly(A) signal
NheI (3632)
BmtI (3636)
AbsI - PaeR7I - PspXI - XhoI (3642)
(3811) BlpI
(4187) SacII
(4603) AsiSI
(4554) RsrII
(4938) PshAI
(5380) MfeI
lac operator
M13 rev
(5740) BspQI - SapI
(5856) PciI
pMT(Hygro)-mCherry-GW
7735 bp
AmpR promoter
AmpR
ori
M13 fwd
MT promoter
mCherry
attR1
lac UV5 promoter
CmR
ccdB
attR2
HygroR
copia promoter
lac promoter
CAP binding site
1000
2000
3000
4000
5000
6000
7000
