# Supplementary material for: Plk4 Is a Novel Substrate of Protein Phosphatase 5
Source: Int J Mol Sci. 2023 Jan 19;24(3):2033. doi: 10.3390/ijms24032033 (PMC9917060; doi:10.3390/ijms24032033)
Supplement: Supplementary file 1 [file ijms-24-02033-s001.zip › Table S1.pdf]

Plk4 is a novel substrate of Protein Phosphatase 5

Edit Ábrahám, Zsuzsánna Réthi-Nagy, Péter Vilmos, Rita Sinka, and Zoltán Lipinszki

Table S1

oligonucleotide primer	sequence	used for
PP5 GW fw	GGGGACAAGTTTGTACAAAAAAGCAGGCTTATCTTCGTCCGAACTGGAAGTACA G	generation of entry clone
PP5 GW rev STOP	GGGGACCACTTTGTACAAGAAAGCTGGGTACTACGCCAGCCAGTTCATTAGGC TG	
PP5 H326N fw	GGTTCATGTTAATGCTTTCGTTGTTGCCGCGCGCCAAG	site-directed mutagenesis
PP5 H326N rev	CTTGGCGCGCGGCAACAACGAAAGCATTAACATGAACC	
PP5 E97Q fw	TGCGAAACCAAAGCTTTGCTGGCGCAAGTGCGC	site-directed mutagenesis
PP5 E97Q rev	GCGCACTTGCGCCAGCAAAGCTTTGGTTTCGCA	
PP5 203-520aa GW fw	GGGGACCACTTTGTACAAGAAAGCTGGGTACTACTGCGGACCCTTGTAGTCAT C	site-directed mutagenesis
PP5 1-202aa GW rev STOP	GGGGACCACTTTGTACAAGAAAGCTGGGTACTACAGCTGCGGACCCTTGTAGT CATC	site-directed mutagenesis
PP5 dsRNA CDS fw	TAATACGACTCACTATAGGGAGACGACATGTACACCAAAGCCATAGAA C	dsRNA generation
PP5 dsRNA CDS rev	TAATACGACTCACTATAGGGAGAAGAGTATTTTGTAGGCAAATTTGCG	
PP5 dsRNA 3'UTR fw	TAATACGACTCACTATAGGGAGAAAAACATACTCCCACACAACACATA C	dsRNA generation
PP5 dsRNA 3' UTR rev	TAATACGACTCACTATAGGGAGAAAGGTTTTTGAACAAAACTGGGGTT	
Kan dsRNA fw	TAATACGACTCACTATAGGGAGAGACAATCTATCGCTTGTATG	dsRNA generation
Kan dsRNA rev	TAATACGACTCACTATAGGGAGAGGAATCGAATGCAACCGGCGC	
PP5 CRDET fw	GCCACCTTCAACATAACC	molecular characterization of PP5 deletion fly strain
PP5 CRDET rev	GCATCCGACTTCTAAAGC	
PP5 Ecl136II fw	GGGAGCTCATGTCTTCGTCCGAACTGG	cloning
PP5 SalI rev	GCGTCGACTACGCCAGCCAGTTCATTAGG	
PP5 Acc65I Myc fw	gcggGTACCGCCACCATGGAGGAGCAGAAGCTGATCTCAGAGGAGGACCTGAT GTCTTCGTCCGAACTGGAAGTAC	cloning
PP5 NotI rev noSTOP	GCGGCGGCCGCCCGCCAGCCAGTTCATTAGG	
HsPP5 GW fw	GGGGACAAGTTTGTACAAAAAAGCAGGCTTAATGGCGATGGCGGAGGGCGAG	generation of entry clone
HsPP5 GW rev STOP	GGGGACCACTTTGTACAAGAAAGCTGGGTATCACATCATTCCTAGCTGCAGCA GC	

Plk4 GW for	GGGGACAAGTTTGTACAAAAAAGCAGGCTTAATGTTATCCAATCGGGCGTTTGG	generation of entry clone
Plk4 GW rev STOP	GGGGACCACTTTGTACAAGAAAGCTGGGTATTAAAGAAGCATGCGATTATAATAAGGCG	
Plk4 ΔKD fw	GGAGAAACAATTGAGGACCCGGCTCACTTGTCTTAC	site-directed mutagenesis
Plk4 ΔKD rev	GTAAGACAAGTGAGCCGGGTCCTCAATTGTTTCTCC	
Plk4 ΔCC fw	GTGATGTCCGAGTACATTATGTTGAAATGCTCAAATGGTGGA	site-directed mutagenesis
Plk4 ΔCC rev	TCCACCATTTGAGCATTTCAACATAATGTACTCGGACATCAC	
Plk4 ΔDRE fw	TTGAGGAAGCACTTGTTGCATAAAGGGATGGCAGAG	site-directed mutagenesis
Plk4 ΔDRE rev	CTCTGCCATCCCTTTATGCAACAAGTGCTTCCTCAA	
Plk4 ΔPB1 fw	GAGTACTGAAGTATGTGACTTTTTCCTTAAGAGATGCCTGTTTG	site-directed mutagenesis
Plk4 ΔPB1 rev	CAAACAGGCATCTCTTAAGGAAAAAGTCACATACTTCAGTACTC	
Plk4 ΔPB2 fw	TGGTAAAAAGCAAAACACCAGCTCAGAGATTGGATGGC	site-directed mutagenesis
Plk4 ΔPB2 rev	GCCATCCAATCTCTGAGCTGGTGTTTTGCTTTTTACCA	
Plk4 ΔPB1-2 fw	CATCCAATCTCTGAGCTTCCTTAAGAGATGCCTGTTTG	site-directed mutagenesis
Plk4 ΔPB1-2 rev	CAAACAGGCATCTCTTAAGGAAGCTCAGAGATTGGATG	
Plk4 ΔPB3 fw	GCTTCCCAGCAATATATTTTGATGGGCGGCTAGC	site-directed mutagenesis
Plk4 ΔPB3 rev	GCTAGCCGCCCATCAAAATATATTGCTGGGAAGC	
Plk4 KD GW fw	GGGGACAAGTTTGTACAAAAAAGCAGGCTTAATGTATGAAGTACAGCACTTGCTGG	site-directed mutagenesis
Plk4 KD GW rev STOP	GGGGACCACTTTGTACAAGAAAGCTGGGTATTACATAATGTACTCGGACATCACA	
Plk4 CC+ DRE GW fw	GGGGACAAGTTTGTACAAAAAAGCAGGCTTACCGGCTCACTTGTCTTACG	site-directed mutagenesis
Plk4 CC+DRE GW rev STOP	GGGGACCACTTTGTACAAGAAAGCTGGGTATTATGGTCCTGAGTTTTCCACAGAT	
Plk4 L1+PB1 GW fw	GGGGACAAGTTTGTACAAAAAAGCAGGCTTAATGCAACAAGTGCTTCCTCAAATACGG	site-directed mutagenesis
Plk4 L1+PB1 GW rev STOP	GGGGACCACTTTGTACAAGAAAGCTGGGTATTATGGTGTTTTGCTTTTTACCAGCCC	site-directed mutagenesis
Plk4 PB1 GW fw	GGGGACAAGTTTGTACAAAAAAGCAGGCTTAATGGATCGCATTTCGGTGCCACCATTG	site-directed mutagenesis
Plk4 PB2 GW fw	GGGGACAAGTTTGTACAAAAAAGCAGGCTTAATGAAAGTCACATACTTCAGTACTC	site-directed mutagenesis
Plk4 PB2 GW rev STOP	GGGGACCACTTTGTACAAGAAAGCTGGGTATTAGGGTTGTACATCTGTTATAGGTC	site-directed mutagenesis
Plk4 L2+PB3 GW fw	GGGGACAAGTTTGTACAAAAAAGCAGGCTTAATGGCTCAGAGATTGGATGGCCTAC	site-directed mutagenesis
Plk4 L2+PB3 GW rev STOP	GGGGACCACTTTGTACAAGAAAGCTGGGTATTATGGAGCTGTCTTCAACTTTAACTG	
Plk4 InF HindIII fw	CGAGCTGTACAAGCTTATGTTATCCAATCGGGCGTTTGG	cloning
Plk4 InF rev noSTOP	CTCTGCCCTCAAGCTTAAGAAGCATGCGATTATAATAAGGC	cloning

HsPlk4 NotI fw	GGGCGGCCGCGATGGCGACCTGCATCGG	cloning
HsPlk4 BamHI rev	GGGGATCCTCAATGAAAATTAGGAGTCGGATTAG	
HsPlk4 ND S285A T289A fw	GTGGAAGACTCAATTGATGCTGGGCATGCCGCAATTTCTACTGCAATTA	site-directed mutagenesis
HsPlk4 ND S285A T289A rev	TAATTGCAGTAGAAATTGCGGCATGCCCAGCATCAATTGAGTCTTCCAC	
Polo GW fw	GGGGACAAGTTTGTACAAAAAAGCAGGCTTAATGGCCGCGAAGCCCGAGGATAAG	generation of entry clones
Polo GW rev Stop	GGGGACCACTTTGTACAAGAAAGCTGGGTATTATGTGAACATCTTCTCCAGC	
